# Correlation between junior high school students’ weight and their family background

**DOI:** 10.3389/fpubh.2025.1638605

**Published:** 2025-09-23

**Authors:** Xiying Zhao, Peng Liu

**Affiliations:** School of Physical Education, Nanjing XiaoZhuang University, Nanjing, China

**Keywords:** middle school students, overweight and obesity, family background, correlation, logistic regression

## Abstract

**Objective:**

The objective of the study was to understand the correlation between weight in junior high school students and the influencing factors within their families in order to provide suggestions for weight management that can improve their health and growth.

**Methods:**

The study included 6,617 junior high school students from the China Education Panel Survey, conducted (CEPS) between 2014 and 2015. This selection was based on the criteria for adolescent overweight and obesity issued by the National Health Commission of the People’s Republic of China, along with the definition of the Chinese family environment used in previous studies. The chi-squared test was performed to compare the association between categorical independent variables and the dependent variable. A binary logistic regression model was utilized to analyze the family environmental factors influencing junior high school students’ weight.

**Results:**

The prevalence of overweight and obesity among junior high school students was 14.39%, with a higher rate observed in boys compared to girls. The multivariate binary logistic regression analysis showed that having a non-agricultural hukou, having parents employed as enterprise managers, and spending more than 2 h gaming on weekends were the primary risk factors for overweight and obesity among middle school students. In contrast, being a child with siblings and strict parental management of screen time were the main protective factors against overweight and obesity.

**Conclusion:**

The influence of family background on overweight and obesity in junior high school students cannot be ignored. The prevention and control of overweight and obesity should start with family health education to improve students’ health and growth.

## 1 Introduction

Weight is closely related to human health, with abnormal weight—particularly overweight and obesity—acting as a key risk factor for chronic diseases (1). The prevalence of overweight and obesity not only imposes heavy financial burden and medical pressure on society but also undermines adolescents’ healthy growth. Notably, these conditions exert numerous negative impacts on their physical and mental health and elevates their risk of developing cardio-cerebrovascular diseases, diabetes, and other chronic diseases in adulthood (2, 3). To fullfil the goals of the Healthy China Initiative (2019–2030), it is necessary to advocate for and promote a civilized and healthy lifestyle. Meanwhile, it is essential to raise public awareness and improve their skills in weight management, as well as in the prevention and control of overweight and obesity, and to effectively advance the prevention and control of chronic diseases at earlier stages (1). Based on this, in June 2024, the National Health Commission, together with 16 other departments, jointly formulated the Implementation Plan for the “Year of Body Weight Management” Activity. In the “Implementation Plan for the ‘Year of Body Weight Management’ Activity,” it is emphasized that the important role of family weight management should be strengthened and weight management should be incorporated into the content of the Healthy Family Construction.

In addition to being related to genetic factors, adolescent weight is more likely caused by acquired factors. In particular, the family environment and the development of living habits have a profound impact on adolescents’ weight. The issue of adolescent weight health has long attracted significant attention and widespread concern worldwide. Extensive research has been conducted on this topic. In China, existing studies have found that adolescent weight is associated with sex, diet, sleep, family background, physical activity, screen time, and parents’ dietary knowledge (4–7). Foreign studies have revealed that parental weight, socioeconomic status (8), screen time (9), lack of general physical activity and outdoor activities (10), and lifestyle (11) are related to adolescent weight health.

In summary, the literature review of previous studies on the influencing factors of adolescent weight health revealed that the majority of studies have relied on local and cross-sectional data; however, there have been limited use of national and longitudinal data. Second, there are still a few previous studies that have explicitly focused on adolescent weight health from the perspective of the family environment. In this context, this study used data from eighth-grade students surveyed during the 2014–2015 academic year by the China Education Panel Survey (CEPS) to understand the relationship between overweight and obesity among junior high school students and the influencing factors within their family environment. Ultimately, the study aims to provide relevant suggestions and a basis for family intervention to prevent and control of weight issues in junior high school students.

## 2 Methods

### 2.1 Data

Participants in this study were selected from the “eighth-grade students” surveyed during the 2014–2015 academic year by the China Education Panel Survey (CEPS). In China’s education system, “junior high school” typically operates on a 3-year system, with students generally aged between 13 and 15 years old. Among them, “eighth-grade students” are second-year students in junior high school, and their actual ages are mainly concentrated in the range of 13 to 14 years old. Ethical approval for the CEPS was provided by the Institutional Review Board at the Renmin University of China, and informed consent from the research participants was obtained before the survey. The survey used a hierarchical, multi-stage probability proportional to size (PPS) sampling method based on the order of county, school, and classroom and aimed to provide nationally representative multi-level basic data support for relevant academic research and policy formulation (12). A total of 28 county-level units (including counties, districts, and cities) were randomly selected from across the country as survey sites. Within these sites, 112 schools and 438 classes were sampled. All students in the selected classes were included in the sample. The survey subjects mainly included students, parents, teachers, and school leaders. This study mainly examined the correlation between the weight of junior high school students and their family environment. For this study, data were selected from the China Education Panel Survey (CEPS), specifically focusing on eighth-grade students during the 2014–2015 academic year. After excluding samples with missing values in key variables, a total of 6,617 student samples were included in the analysis.

### 2.2 Study design

Trained investigators used “the Questionnaire for Eighth-grade Students in the China Education Panel Survey (CEPS) during the 2014–2015 Academic Year” to conduct the survey among the students. The survey covered multiple dimensions, including students’ basic information, growth experiences, physical and mental health, learning in the school, extracurricular activities, and family educational environment. According to the research requirements, this study ultimately included three parts: sociodemographic characteristics, overweight and obesity status, and family environment factors. Data on the students’ height and weight were self-reported by the students at the time of the survey. The distinction between overweight and obesity was made based on the BMI cutoff values set respectively according to gender and age differences in the National Health Standard (13) “Screening for Overweight and Obesity in School-Age Children and Adolescents” (WS/T568-2018). The details are shown in Table 1. Among the variables, age was calculated in half-year units, and chronological age was uniformly used. Chronological age is calculated by subtracting the date of birth from the survey date, referring to the total number of full years experienced from birth to the time of calculation. In this standard, it was measured in half-year units. Individuals with a BMI greater than or equal to the “overweight” cutoff value but less than the “obesity” cutoff value for the corresponding sex and age group were classified as overweight. Those with a BMI greater than or equal to the “obesity” cutoff value for the corresponding sex and age group were classified as obese.

**Table 1:** BMI cutoff values for screening overweight and obesity in the adolescents by sex and age (kg/m^2^).

Age (years old)	Male individuals		Female individuals	
	Overweight	Obesity	Overweight	Obesity
13.0~	21.4	25.2	22.2	25.0
13.5~	21.9	25.7	22.6	25.6
14.0~	22.3	26.1	22.8	25.9
14.5~	22.6	26.4	23.0	26.3

Referring to the studies conducted by Li Dandan (14) and Yuan Fan (15), this study divided the family environment into three dimensions: the basic growth environment of the family, the family behavioral educational environment, and the family dietary environment. The basic growth environment of the family included four indicators: household registration type, whether the student is an only child, family economic situation, and the occupational stratification of parents. Referring to the classification methods of the occupational stratification of parents in Hong Yanbi’s (16) research, this study selected the parent with the higher occupational stratification, among the two parents, as the independent variable. The family behavioral educational environment included five indicators: parents’ management of children’s Internet usage time, parents’ management of children’s TV watching time, children’s sleep time, individuals’ daily online time, and individuals’ daily game-playing time and TV-watching time on weekends. The family dietary environment included two indicators: whether the family’s daily diet often consists of grilled, fried, and Western fast food and whether the family’s daily diet includes sugary and carbonated beverages.

### 2.3 Statistical analysis

Statistical analysis was conducted using the social statistics software Stata 13.0. Since the dependent variable was whether one was overweight or obese, which is a binary variable, the chi-squared test was used to compare the association between categorical independent variables and the dependent variable. A binary logistic regression model was utilized to analyze the family environmental factors influencing the junior high school students’ weight, with the examination level set at α =0.05.

## 3 Results

### 3.1 Basic situation of the junior high school students’ weight

Variables were screened according to the research objectives. A total of 6,617 junior high school students were included in the analysis sample for this study. There were 952 students who were overweight or obese, accounting for 14.39%. Among the 6,617 students, there were 3,396 male students and 3,221 female students. The number of male students who were overweight or obese was 632 (18.61%), and the number of female students who were overweight or obese was 320 (9.93%). The overweight and obesity rate in the male students was higher than that in the female students (χ^2^ = 101.007, *p* < 0.01), as shown in Table 2.

**Table 2:** Comparison of basic information on overweight detection rates among the junior high school students [*n* (%)].

Sex	Total	The situation of weight	
		Non-overweight and obese	Overweight and obese
Male	3,396	2,764 (81.54)	632 (18.61)
Female	3,221	2,901 (90.22)	320 (9.93)
*χ^2^*		101.01	
*p*		<0.01	

### 3.2 Univariate difference analysis

In this study, the weight of the junior high school students was categorized into the non-overweight and non-obese group and the overweight and obese group. After analysis, it was found that the samples in both groups were affected by factors such as household registration type, whether they were the only child, family economic conditions, father’s/mother’s occupational class, parents’ management of Internet usage time, sleep duration, daily duration of Internet surfing and game playing on weekends, and whether they often drink sugary beverages (*p* < 0.05). However, parents’ management of the duration of TV viewing, daily TV viewing duration on weekends, and whether they often eat fried and grilled foods had no noticeable impact on the prevalence of overweight and obesity (*p* > 0.05), as shown in Table 3.

**Table 3:** Single-factor analysis of the influence of family factors on the junior high school students’ weight.

Influencing Factors	Classification	Total	Overweight and obese [*n* (%)]	*χ^2^*	*p*
Household registration type	Rural	3,478	410 (11.79)	40.20	0.00**
	Non-rural	3,139	542 (17.27)		
Only child	Only child	3,001	549 (18.29)	68.05	0.00**
	Non-only child	3,616	403 (11.14)		
Family economic conditions	Extremely difficult	164	18 (10.98)	31.53	0.00**
	Relatively difficult	766	69 (9.01)		
	Moderate	4,880	712 (14.59)		
	Relatively wealthy	759	143 (18.84)		
	Extremely wealthy	48	10 (20.83)		
Father’s / Mother’s occupational class	Lower class	1926	203 (10.54)	33.55	0.00**
	Middle class	3,138	494 (15.74)		
	Upper class	1,553	253 (16.42)		
Parents’ management of internet use duration	Do not care	432	79 (18.29)	11.22	0.00**
	Care, but not strictly	2,349	363 (15.45)		
	Care strictly	3,836	510 (13.30)		
Parents’ management of the duration of TV viewing	Do not care	759	128 (16.86)	5.52	0.06
	Care, but not strictly	3,285	477 (14.52)		
	Care strictly	2,573	347 (13.49)		
Sleep duration	<= 6 h	368	67 (18.21)	7.02	0.03*
	6 to 8 h	3,450	510 (14.78)		
	> = 8 h	2,799	375 (13.40)		
Daily TV viewing duration on weekends	None	762	112 (14.70)		
	Less than 2 h	2,303	313 (13.59)	6.76	0.24
	2 to 4 h	2,138	308 (14.41)		
	4 to 6 h	890	126 (14.16)		
	6 to 8 h	252	48 (19.05)		
	> = 8 h	272	45 (16.54)		
Daily duration of internet surfing and game playing on weekends	None	1,381	160 (11.59)		
	<2 h	2,211	279 (12.62)	34.38	0.00**
	2 to 4 h	1727	279 (16.16)		
	4 to 6 h	687	116 (16.89)		
	6 to 8 h	270	51 (18.89)		
	> = 8 h	341	67 (19.65)		
Whether one eats fried, barbecued, and puffed food	Never	301	44 (14.62)		
	Seldom	2,392	331 (13.84)	4.81	0.31
	Sometimes	2,953	452 (15.31)		
	Often	878	115 (13.10)		
	Always	93	10 (10.75)		
Whether one drinks sugary and carbonated beverages	Never	247	57 (23.08)		
	Seldom	2,147	298 (13.88)	18.81	0.00**
	Sometimes	2,813	416 (14.79)		
	Often	1,249	159 (12.73)		
	Always	161	22 (13.66)		

“*” *p*<0.05, “**” *p*<0.01.

### 3.3 Logistic regression analysis of the influencing factors

On the basis of the single-factor analysis, whether being overweight or obese was set as the dependent variable (non-overweight and non-obese = 0; overweight and obese = 1), and the factors related to the family environment that influenced overweight and obesity were considered independent variables. By selecting the factors related to the family environment that had a noticeable impact and then putting these factors into the binary logistic regression analysis, the study went on to explore the influence of the factors related to the family environment on the body weight of junior high school students. The specific variable assignments are shown in Table 4.

**Table 4:** Value assignment for the relationship between the weight of junior high school students and family environmental factors.

Influencing factors	Value assignment
Household registration type	Rural = 0; Non-rural = 1
Only child	only child = 0; Non-only child = 1
Family economic conditions	Extremely difficult = 1; Relatively difficult = 2; Moderate = 3; Relatively wealthy = 4; Extremely wealthy = 5
Father’s/Mother’s occupational class	Lower class = 1; Middle class = 2; Upper class = 3
Parents’ management of internet use Duration	Do not care = 1; Care, but not strictly = 2; Care strictly = 3
Sleep duration	<=6 h = 1; 6 to 8 h = 2; > 8 h = 3
Daily duration of internet surfing and game playing on weekends	None = 1; Less than 2 h = 1; 2 to 4 h = 3; 4 to 6 h = 4; 6 to 8 h = 5; > 8 h = 6
Whether one drinks sugary and carbonated beverages	Never = 1; Seldom = 2; Sometimes = 3; Often = 4; Always = 5

Logistic regression analysis was conducted with the junior high school students’ overweight and obesity status as the dependent variable. The results showed that having a non-rural household registration, having parents with middle-level occupations, and spending more than 2 h a day playing online games on weekends were risk factors for the occurrence of overweight and obesity. In contrast, being a non-only child, parents’ management of online time, and consumption of sugary beverages were protective factors for the occurrence of overweight and obesity. However, family economic conditions and sleep duration had no noticeable impact on the occurrence of overweight and obesity. The details are shown in Table 5.

**Table 5:** Logistic regression analysis of the relationship between the body weights of junior high school students and family environmental factors.

Influencing factors	Classification	*β*	S. E.	*p*	OR	95% CI
Household registration type	Rural *					
	Non-rural	0.21	0.08	0.01	1.24	1.05 ~ 1.45
Only child	Only child *					
	Non-only child	−0.44	0.08	0.00	0.65	0.55 ~ 0.75
Family economic conditions	Extremely difficult *					
	Relatively difficult	−0.20	0.28	0.49	0.82	0.47 ~ 1.43
	Moderate	0.10	0.26	0.69	1.11	0.67 ~ 1.84
	Relatively wealthy	0.32	0.28	0.25	1.37	0.80 ~ 2.36
	Extremely wealthy	0.45	0.44	0.32	1.56	0.65 ~ 3.73
Father’s / Mother’s occupational class	Lower class*					
	Middle class	0.20	0.09	0.04	1.22	1.01 ~ 1.48
	Upper class	0.06	0.12	0.63	1.06	0.84 ~ 1.34
Parents’ management of internet use duration	Do not care*					
	Care, but not strictly	−0.28	0.14	0.04	0.76	0.57 ~ 0.99
	Care strictly	−0.34	0.14	0.02	0.72	0.54 ~ 0.94
Sleep duration	<= 6 h					
	6 to 8 h	−0.19	0.15	0.21	0.83	0.62 ~ 1.11
	>8 h	−0.23	0.15	0.13	0.79	0.59 ~ 1.07
Daily duration of internet surfing and game playing on weekends	None*					
	<2 h	−0.04	0.11	0.74	0.96	0.79 ~ 1.19
	2 to 4 h	0.27	0.11	0.02	1.30	1.05 ~ 1.63
	4 to 6 h	0.35	0.14	0.01	1.41	1.08 ~ 1.86
	6 to 8 h	0.50	0.18	0.01	1.65	1.15 ~ 2.36
	> = 8 h	0.53	0.17	0.00	1.69	1.21 ~ 2.38
Whether one drinks sugary and carbonated beverages	None*					
	Seldom	−0.63	0.17	0.00	0.53	0.38 ~ 0.74
	Sometimes	−0.64	0.17	0.00	0.53	0.38 ~ 0.73
	Often	−0.94	0.18	0.00	0.39	0.28 ~ 0.56
	Always	−1.06	0.28	0.00	0.35	0.20 ~ 0.60
Pseudo R^2^	0.03					
Log likelihood	−2644.53					
Chi2	162.42					

“*” represents the control group.

Specifically, under the condition that other family factors are the same, the probability of overweight and obesity among the junior high school students with a non-rural household registration was 24% higher than that among those with a rural household registration. The probability of overweight and obesity among the junior high school students whose parents are in middle-level occupations was 22% higher than that among those whose parents are in bottom-level occupations. The probability of overweight and obesity among the junior high school students who spend more than 2 h per day on online games on weekends was 30% ~ 69% higher than that among those who do not play online games on weekends. The probability of overweight and obesity among the junior high school students who are non-only children was 35% lower than that among the only children; and the probability of overweight and obesity among the junior high school students whose parents strictly manage their Internet usage time was 24% ~ 28% lower than that among those whose parents do not impose such management. In addition, the probability of overweight and obesity among the junior high school students who consume sugary drinks was 47% ~ 65% lower than that among those who do not consume such drinks.

## 4 Discussion

This study examined the weight status of junior high school students and its correlation with family environmental influencing factors, using data from 6,617 eighth-grade students surveyed during the 2014–2015 academic year by the China Education Panel Survey (CEPS). It was found that the prevalence of overweight and obesity among the junior high school students was 14.39%. Among them, the prevalence of overweight and obesity in the male students was 18.61% and in the female students was 9.93%. The incidence of overweight and obesity in the male students was approximately twice that in the female students. This is relatively similar to previous findings by Gao Li wang (17) and Wang Yun (18) on the prevalence trend of overweight and obesity among junior high school students. The research further explored the correlation between the incidence of overweight and obesity among junior high school students and the family environment.

### 4.1 Correlation between family-based growth environments and the weight of junior high school students

Junior high school students with non-agricultural household registration, those who are only children, or those whose parents are in the middle of their careers are more likely to be overweight or obese. Specifically, the incidence of overweight and obesity among the non-agricultural household registration junior high students was higher than that among the agricultural household registration junior high students. The reason may be that the economic development in towns is better and parents in urban families generally have relatively higher educational expectations for their children. As a result, the students’ learning burden increases, and they spend more time on their schoolwork and have insufficient time for extracurricular physical activities (19).

It may also be related to the fact that the urban food environment is more likely to promote high-energy intake, directly increasing the risk of overweight. Meanwhile, urban spatial design and convenient transportation have reduced physical activity levels. Being an only child is a risk factor for overweight and obesity. This finding is in line with the research conclusions of Ren Shi (20) and others. The main reason may be that only-child parents pay more attention to their children. In Chinese one-child families, the “421 structure” (four grandparents + two parents + one child) is prevalent. The “over-protection” in intergenerational parenting may reduce children’s opportunities for independent physical activities. For example, parents or grandparents may drive their children to and from school, instead of letting them walk, or restrict their outdoor adventurous activities. In contrast, in non-only-child families, daily interactions among siblings, such as outdoor games and physical competitions, can naturally increase the amount of physical activity. Moreover, only children, lacking such peer interactions, are more likely to rely on electronic devices for entertainment, which prolongs their screen time and leads to increased sedentary behavior (21).

Children from families where parents are in the middle of their careers are at a higher risk of being overweight or obese. The main reason may be that families in the middle of the profession usually occupy a middle position in society in terms of economic and social resources. To maintain the family’s social status or expect their children to move to a higher social class, they tend to be more competitive in educating their children, which increases the children’s learning burden and reduces relative physical activity time (19).

### 4.2 Correlation between family behavioral education and the weight of junior high school students

The family is the first place where children learn socialization, and they spend most of their time there before entering high school. Parents’ educational methods provide the environmental soil for children’s growth, and this environment shapes all kinds of behaviors (22). Families that regulate their children’s online time have lower rates of overweight and obesity among middle school students compared to families that do not pay attention to their children’s online time. Middle school students who spend more than 2 h online or playing games on weekends were found to have a higher risk of being overweight or obese, which is similar to the findings of Shi Ping (23) and others. Therefore, parents should arrange appropriately the time middle school students spend surfing the Internet and playing games, reduce the usage time of electronic devices and the Internet, and guide their children to have healthy entertainment and leisure activities.

### 4.3 Correlation between family dietary characteristics and the weight of junior high school students

Previous studies (24–26) have found that drinking sugary beverages is a risk factor for the occurrence of overweight and obesity among middle school students. However, this study did not report this finding. This may be related to the heterogeneity of the samples used in previous studies, as the samples in the previous studies (25, 26) also included children under 13 years old. Admittedly, due to the cross-sectional nature of this study, there may be a risk of “reverse causation bias.” That is, junior high school students who are overweight or obese may have had their intake of sugar-sweetened beverages restricted by their parents or themselves (out of concern that their weight will increase further), resulting in a superficial association between “high intake of sugar-sweetened beverages” and “normal weight” in the data, rather than a true causal relationship.

### 4.4 Limitations

Although the study reveals a correlation between the weight of junior high school students and the family environment, there are still some limitations. First, there is a limitation regarding the timeliness of the data. We acknowledge that changes in social development, the increased use of digital media, and changes in public policies may have influenced these findings. Second, the study was based on cross-sectional data and revealed only the correlation between the weight of junior high school students and their family environment; however, it cannot determine a causal relationship. Third, the lack of analyses conducted separately for male and female students may affect the applicability of the conclusions in implemented public health education programs. Fourth, questionnaire surveys are subject to a certain degree of recall bias, including issues with the definition of variables, biases in subjective reporting, and the omission of relevant variables, among others. When reporting indicators such as height and weight, there may be subjective information bias. Finally, this study was conducted within the context of Chinese education, which may make it difficult to generalize the findings to other countries or regions.

## 5 Conclusion

The family environment is essential for the healthy growth of junior high school students, and families should prioritize weight management for these adolescents. Parents are not only the supervisors of their children’s behaviors but also the first teachers guiding their children toward a healthy life. Therefore, society should strengthen the promotion of educational theories on family health and share knowledge about healthy living and lifestyles with the public. Parents should regulate the amount of time their children spend using electronic devices and playing Internet games during their free time. It is beneficial to reduce the time children spend sitting still and being inactive. By doing this, the risk of children becoming overweight or obese is lowered, contributing to their overall healthy growth.

Meanwhile, if updated follow-up data from the China Education Panel Survey (CEPS) or other high-quality new datasets become available in the future, we plan to conduct further research to re-examine and expand these correlations. This will, in turn, enhance their relevance to contemporary adolescents. Furthermore, we look forward to exploring the causal relationship through longitudinal tracking data in the future.

## Data Availability

The data analyzed in this study is subject to the following licenses/restrictions: the data of the China Education Panel Survey (CEPS) requires application for use. Requests to access these datasets should be directed to ceps@nsrcruc.org; http://ceps.ruc.edu.cn/.
